# Grip strength modifies the association between estimated glomerular filtration rate and all-cause mortality

**DOI:** 10.1093/ndt/gfz140

**Published:** 2019-07-16

**Authors:** Per-Ola Sundin, Ruzan Udumyan, Katja Fall, Scott Montgomery

**Affiliations:** 1 Clinical Epidemiology and Biostatistics, School of Medical Sciences, Örebro University, Örebro, Sweden; 2 Medical Epidemiology and Biostatistics, Karolinska Institutet, Stockholm, Sweden; 3 Clinical Epidemiology Division, Department of Medicine, Karolinska University Hospital Solna, Karolinska Institutet, Stockholm, Sweden; 4 Department of Epidemiology and Public Health, University College, London, UK

Chronic kidney disease (CKD) with reduced glomerular filtration rate (GFR) represents a high-magnitude increased risk for cardiovascular disease (CVD) and all-cause mortality (ACM) [1]. The main non-GFR determinant of the endogenous filtration marker creatinine is its production rate in muscle [2]. Stratification of GFR by a marker of muscle function (grip strength) associated with muscle mass [3] may remove heterogeneity, providing a more accurate marker of mortality risk.

This study evaluates whether grip strength identifies ACM risk associated with estimated GFR (eGFR) from serum creatinine in a subsample of the UK Household Longitudinal Survey (UKHLS) [4–6]. Of the eligible participants, 10 900 had complete data, with an eGFR of 15–120 mL/min/1.73 m^2^ body surface area (BSA) and followed for up to 4–5 years [7].

eGFR was determined using the Chronic Kidney Disease Epidemiology Collaboration equation [8] and grip strength was standardized by age and sex. Baseline diagnoses were self-reported. ACM was reported by the diseased individual’s household or identified through systematic enquiries in the event of non-contact; otherwise participants were classified as alive.

All procedures were in accordance with the Helsinki Declaration of 1975 on ethical principles for medical research involving human subjects, as revised in 2013 [9].

Associations between eGFR and ACM were evaluated using logistic regression (due to the wave structure of the data) adjusting for age, sex, ethnicity, body mass index (BMI), smoking and self-reported pre-existing diagnoses of CVD (ischaemic heart disease or stroke), diabetes and hypertension. The linearity of the relationship between the log odds of mortality and the continuous variables eGFR, age and BMI was assessed by applying the multivariable fractional polynomial method [10]. Odds ratios (ORs) for ACM were calculated for the following categories of eGFR: median 37.5 (range 30–44), 52.5 (45–60), 75 (60–89), 97.5 (90–104) and 112.5 (105–119) mL/min/1.73 m^2^ BSA using 90 mL/min/1.73 m^2^ BSA as a reference. Sparsely populated categories at the extremes of the distribution are not included. Effect modification by grip strength was evaluated by adding the main effects and the eGFR–grip strength multiplicative interaction term to the adjusted model. The association between eGFR and ACM was further estimated by stratifying the adjusted model into thirds of the distribution of grip strength. Sensitivity analyses included additional adjustment for chronic obstructive pulmonary disease, cancer and congestive heart failure at baseline. Further sensitivity analysis included eGFR values outside the 15–120 mL/min/1.73 m^2^ BSA range. Additional information is available in the Supplementary data Methods.

Mortality was recorded for 2.48% (270/10900). Standardized grip strength is not correlated with eGFR (Pearson’s correlation coefficients −0.04–0.01). The multiplicative interaction between grip strength and eGFR for mortality risk is statistically significant (P = 0.04). Characteristics of the study population and adjusted OR for ACM calculated at specified eGFR values are presented in Table 1. In the entire sample, the corresponding unadjusted OR decrease linearly from 12.8 [95% confidence interval (CI) 8.90–16.67] at eGFR 37.5 mL/min/1.73 m^2^ BSA to 0.34 (0.30–0.73) at eGFR 112.5 mL/min/1.73 m^2^ BSA compared with eGFR 90 mL/min/1.73 m^2^ BSA. The adjusted OR for the lowest third of grip strength compared with the highest associated with ACM is 1.77 (95% CI 1.28–2.46). The distribution of eGFR values is illustrated in Supplementary data, Figure S1. Results for all covariates are presented in Supplementary data, Table S1. Sensitivity analyses did not indicate notable changes in associations (Supplementary data, Tables S2 and S3).

**Table 1:** Characteristics of the study population and associations of eGFR with ACM

		Grip strength^a^			
Characteristics	Total	Lowest third	Middle third	Highest third	P-value
	(*n* = 10 900)	(*n* = 3637)	(*n* = 3660)	(*n* = 3603)	
Characteristics of the study population^b^					
Age (years), mean (SD)	53.50 (15.70)	53.77 (16.23)	53.22 (15.55)	53.51 (15.30)	0.32
Female sex, *n* (%)	5989 (54.9)	1990 (54.7)	2053 (56.1)	1946 (54.0)	0.19
Ethnicity, *n* (%)					<0.001
White UK	10 067 (92.4)	3329 (91.5)	3361 (91.8)	3377 (93.7)	
Afro-Caribbean	87 (0.8)	20 (0.5)	36 (1.0)	31 (0.9)	
Other	746 (6.8)	288 (7.9)	263 (7.2)	195 (5.4)	
Serum creatinine (μmol/L)	75 (65–86)	74 (64–85)	74 (64–86)	76 (88–87)	<0.001
BMI	27.4 (24.5–30.9)	27.0 (24.0–30.7)	27.2 (24.2–30.4)	28.1 (25.1–31.4)	<0.001
Smoking, *n* (%)					<0.01
Never regular smoker	4431 (40.7)	1499 (41.2)	1550 (42.3)	1382 (38.4)	
Former regular smoker	4429 (40.6)	1431 (39.3)	1444 (39.5)	1554 (43.1)	
Current smoker	2040 (18.7)	707 (19.4)	666 (18.2)	667 (18.5)	
Diabetes, *n* (%)	810 (7.4)	361 (9.9)	237 (6.5)	212 (5.9	<0.001
CVD, *n* (%)	621 (5.7)	268 (7.4)	182 (5.0)	171 (4.7)	<0.001
Hypertension, *n* (%)	2089 (19.2)	741 (20.4)	674 (18.4)	674 (18.7)	0.07
eGFR categories (mL/min/1.73 m^2^), *n* (%)					<0.001
15–29	38 (0.3)	24 (0.7)	5 (0.1)	9 (0.2)	
30–44	156 (1.4)	72 (2.0)	45 (1.2)	39 (1.1)	
45–59	561 (5.1)	194 (5.3)	184 (5.0)	183 (5.1)	
60–89	4564 (41.9)	1402 (38.5)	1515 (41.4)	1647 (45.7)	
90–104	3541 (32.5)	1180 (32.4)	1197 (32.7)	1164 (32.3)	
105–119	2040 (18.7)	765 (21.0)	714 (19.5)	561 (15.6)	
Adjusted OR (95% CI) for ACM^c^					
eGFR categories [median (IQR)]					
37.5 (30–44)	1.68 (1.09–2.61)	2.24 (1.39–3.61)	0.94 (0.38–2.29)	0.83 (0.30–2.34)	
52.5 (45–59)	1.07 (0.77–1.50)	1.50 (1.18–1.91)	0.96 (0.51–1.81)	0.88 (0.42–1.83)	
75.0 (60–89)	0.86 (0.71–1.04)	1.12 (1.05–1.20)	0.98 (0.76–1.27)	0.95 (0.71–1.27)	
90.0	Ref	Ref	Ref	Ref	
97.5 (90–104)	1.18 (1.03–1.35)	0.96 (0.93–0.98)	1.01 (0.89–1.15)	1.03 (0.89–1.19)	
112.5 (105–119)	1.95 (1.20–3.17)	0.89 (0.83–0.95)	1.03 (0.70–1.50)	1.08 (0.70–1.68)	

a Grip strength standardized by age and sex.

b Values are presented as median [interquartile range (IQR)] unless stated otherwise. Categorical measures are compared using Pearson’s chi-squared test and continuous measures using the Kruskal–Wallis test, with the exception of age, which was compared using analysis of variance.

c Adjusted for age, sex, ethnicity, BMI, smoking and self-reported pre-existing diagnoses of CVD, diabetes and hypertension. Stratified into thirds of the distribution of grip strength. Non-linearity of the association for eGFR, age and BMI was modelled as specified in the Supplementary data, Methods.

In this large general population cohort, eGFR has a U-shaped association with increased ACM risk in adjusted models. Following stratification into thirds of the grip strength distribution, a statistically significant association between low eGFR and increased ACM risk is present only for the lowest grip strength (there is no elevated risk in the two higher thirds). These findings suggest that associations between lower eGFR and increased ACM are driven disproportionally by individuals with low grip strength. Testing grip strength may be a convenient and inexpensive way to improve the stratification of ACM risk based on eGFR.

Low grip strength may indicate sarcopenia [3], which is associated with increased mortality risk in patients with CKD independent of GFR [11]. Recent papers have established an association between low grip strength and ACM for dialysis patients where variation in GFR is small [12], unlike in our study, which has much greater variation. However, as this study identified a ‘multiplicative’ interaction of grip strength with eGFR for mortality risk, the additional independent risk associated with sarcopenia cannot explain our results; neither can lower creatinine production in sarcopenia. Low grip strength is associated with changes in metabolic, inflammatory and hormonal systems potentially relevant to the adverse effects of CKD [13–15].

Our study has several major strengths, including the large general population sample and the use of flexible modelling. No previous study that we are aware of evaluated the effect modification by grip strength for the association between low eGFR and ACM.

Our study also has potential limitations. Due to the distribution of our data, we estimated ACM risk associated with a mild or moderate reduction of eGFR, but this is of major public health importance. Our data do not include measured GFR or a specific measure of muscle mass, nor do they allow estimation of risk of progression of CKD to end-stage renal disease. The relatively short follow-up may not allow some low-risk groups to be represented fully in the results. Morbidity associated with low eGFR may not be as pronounced in our general population sample as in a clinical population, as individuals living in institutions or with a medical history indicating non-eligibility for taking blood samples are not included. Mortality may be somewhat underestimated, as it is obtained from other household members or by enquiries by the researchers. UKHLS data have not been linked to national health registers, so diagnoses are self-reported and cause-specific mortality cannot be evaluated. For the above reasons, ACM is detected with reduced sensitivity and mortality among the people in the poorest health may be underestimated. These potential limitations do not invalidate the potential usefulness of combining grip strength and eGFR to estimate mortality risk.

Grip strength may be used to improve estimates of eGFR-related ACM risk. To refine this further, studies should include both eGFR and measured GFR, as well as measures of muscle strength or muscle mass.

## Supplementary Material

gfz140_Supplementary_Data
