# Erratum to: Grip strength modifies the association between estimated glomerular filtration rate and all-cause mortality

**DOI:** 10.1093/ndt/gfz215

**Published:** 2019-11-09

**Authors:** Per-Ola Sundin, Ruzan Udumyan, Katja Fall, Scott Montgomery

Grip strength modifies the association between estimated glomerular filtration rate and all-cause mortality, *Nephrol Dial Transplant* 2019; gfz140. doi: 10.1093/ndt/gfz140

In the original article, there were errors in Table 1. The number for the row ‘Ages (years), mean (SD)’ and the column ‘Highest third’ was incorrect and the number for the row ‘Ethnicity, Other’ and the column ‘Highest third’ was missing. These have now been corrected.

