# Supplementary material for: Grip strength modifies the association between estimated glomerular filtration rate and all-cause mortality
Source: Nephrol Dial Transplant. 2019 Jul 16;34(10):1799–801. doi: 10.1093/ndt/gfz140 (PMC6775473; doi:10.1093/ndt/gfz140)
Supplement: gfz140_Supplementary_Data [file gfz140_supplementary_data.docx]

**Supplementary methods**

The British Household Panel Survey (BHPS) established in 1999-2001 has followed the members of approximately 9,000 UK households up to 2008 when the study population was absorbed by the new UK Household Longitudinal Survey (UKHLS). This large national representative longitudinal panel study is following members of approximately 40,000 households in the UK since 2009-2010. Annual interviews are performed in overlapping 2-year waves and BHPS participants were invited to join the study at wave 2 (2010-2012). Trained nurses collected anthropometric and health measures including non-fasting blood samples, on average 5 months after the wave 2 interview for non-BHPS participants and the wave 3 interview for the BHPS sample [1]. Non-pregnant respondents aged 16 years or older, living in England, Scotland or Wales, who completed their interview in English were eligible for nurse visits, and among 35,937 eligible respondents 20,700 participated. Some 1,579 individuals who volunteered at the nurse visit that they were HIV positive, had hepatitis B or C, had a bleeding or clotting disorder, took anti-clotting medication excluding aspirin or had ever had a seizure, were not eligible to give blood. Another 4,688 respondents refused to give blood. Blood samples were successfully obtained and at least one blood-based biomarker determined in 13,107 individuals. After first excluding participants with missing data on any of the measures included in our analysis (n=1,476) and finally excluding participants with eGFR values outside the range of 15-120 ml/min/1.73 m^2^ body surface area (n=731), a total of 10,900 respondents were included in the final analytical sample. The range of eGFR values was chosen to exclude individuals with end-stage renal disease and to avoid estimating GFR in the highest range where precision is known to be low [2]. The study population was followed for 4-5 years, from the baseline nurse health assessment in 2010-2013 to the seventh wave in 2015-2017. All analyses were adjusted for at which wave participants entered the study.

The UKHLS survey was approved by the Ethics Committee of the University of Essex and the National Research Ethics Service Oxfordshire REC A (10/H0604/2; 10/H0604/62; 10/H0604/70).

Procedures for obtaining the anthropometric and health measures have been described in detail by McFall et al [1]. Serum creatinine was determined using an enzymatic method on the Roche P module analyser. Inter and intra assay coefficients of variation were less than 4%. The creatinine assay used calibrators traceable to isotope dilution mass spectrometry (IDMS). Grip strength was measured in the dominant hand in standing position with upper arm against the trunk and forearm at a right angle to the upper arm. The maximum reading from three attempts was recorded using a Smedley’s dynamometer. Standard deviation scores of grip strength were created within each sex and 10-year age groups starting at age 16 (ages above 86 years collapsed due to low numbers) and these were combined to form a standardised measure. Body weights above 130 kg were self-reported due to imprecision of the scale in that range.

Self-reported baseline diagnoses were identified in the wave that preceded the nurse health assessment; wave 2 for non-BHPS participants and the wave 3 interview for the BHPS sample. Chronic obstructive pulmonary disease (COPD) was identified as self-reported chronic bronchitis or emphysema. Glycated haemoglobin (HbA1c) was measured from whole blood using HPLC cation exchange on a Tosoh G8 analyser. Intra and inter assay coefficients of variation were less than 4%. Baseline diabetes mellitus was defined as self-reported disease or a HbA1c value ≥48 mmol/mol. Data on smoking were not collected in wave 3 and therefore smoking status was determined at wave 2 for all participants. Smoking was classified as never regular smoker, ex-smoker or current smoker. Ethnicity was classified as white UK, Afro-Caribbean and other.

P-values were two-sided and considered statistically significant at the 0.05 level. All statistical analyses were conducted using STATA 14 (Stata Corp., College Station, TX.)

**Transformations of continuous variables**

Transformations of continuous variables applied in the multivariable fractional polynomial (MFP) analysis were selected from a fixed set including exponentiating the variable by the power of -2, -1, -0.5, 0, 0.5, 1, 2, 3 were 1 indicate no transformation and 0 is equal to logaritmic transformation [3, 4]. The MFP analysis evaluated both first-degree fractional polynomial (FP1) functions and second-degree fractional polynomial (FP2) functions using combinations of two powers to transform the variable. No data-driven selection of variables to include in the models was performed; all pre-specified variables were included. The level of significance for transformation of estimated glomerular filtration rate was set to 5% and for transformation of potential confounders a 20% level of significance was applied. Age was modelled as linear in unadjusted and all adjusted models. Body mass index was modelled as linear in the unadjusted model and in the adjusted models for the middle and highest strata of standardized grip strength. In the adjusted model before stratification and in the model for the lowest stratum of grip strength, body mass index was modelled using a FP2 function with powers (1,1). Estimated glomerular filtration rate was modelled as non-linear in the adjusted model before stratification and in the adjusted model for the lowest stratum of grip strength using a FP1 function with power (-1). In sensitivity analyses not excluding individuals with estimated glomerular filtration rate outside the range of 15-120 ml/min/1.73 m^2^ body surface area, estimated glomerular filtration rate was modelled using a FP1 function with power (-0.5) in the adjusted model for the lowest stratum of grip strength. In sensitivity analyses adding additional adjustment for pre-existing cancer, pre-existing chronic obstructive pulmonary disease or pre-existing congestive heart failure, the same transformations as in the main analyses were selected.

**REFERENCES**

1. McFall S, Petersen J, Kaminska O, Lynn P. (2014). Understanding Society – UK Household Longitudinal Study: Waves 2 and 3 Nurse Health Assessment, 2010-2012, Guide to Nurse Health Assessment. Colchester: University of Essex. 2014

2. Gaspari F, Ruggenenti P, Porrini E*, et al.* The GFR and GFR decline cannot be accurately estimated in type 2 diabetics. Kidney International 2013;84:164-173

3. Royston P, Ambler G, Sauerbrei W. The use of fractional polynomials to model continuous risk variables in epidemiology. Int J Epidemiol 1999;28:964-974

4. Sauerbrei W, Royston P, Binder H. Selection of important variables and determination of functional form for continuous predictors in multivariable model building. Stat Med 2007;26:5512-5528

| **Supplementary table 1.** Associations between covariates and all-cause mortality. | | | | | | | | | | | | |
| --- | --- | --- | --- | --- | --- | --- | --- | --- | --- | --- | --- | --- |
|  |  | **Unadjusted^a^** | |  | **Adjusted^ab^ model stratified by grip strength** | | | | | | | |
|  |  |  |  |  | **Lowest third** | |  | **Middle third** | |  | **Highest third** | |
| **Covariate** |  | **OR** | **95% CI** |  | **OR** | **95% CI** |  | **OR** | **95% CI** |  | **OR** | **95% CI** |
| Age (years) |  | 1.11 | 1.10-1.12 |  | 1.10 | 1.08-1.13 |  | 1.12 | 1.09-1.15 |  | 1.10 | 1.07-1-14 |
| Male sex |  | 1.69 | 1.32-2.16 |  | 1.82 | 1.21-2.74 |  | 1.09 | 0.68-1.76 |  | 1.85 | 1.06-3.24 |
| Ethnicity |  |  |  |  |  |  |  |  |  |  |  |  |
| White UK |  | REF |  |  | REF |  |  | REF |  |  | REF |  |
| Afro-Caribbean |  | 1.30 | 0.41-4.15 |  | 1.90 | 0.34-10.58 |  | 2.39 | 0.25-22.80 |  | -^c^ |  |
| Other |  | 0.36 | 0.17-0.76 |  | 0.26 | 0.06-1.20 |  | 0.37 | 0.09-1.62 |  | 1.52 | 0.45-5.19 |
| Body mass index |  |  |  |  |  |  |  |  |  |  |  |  |
| 18.5 |  | 1.04 | 0.90-1.21 |  | 2.82 | 1.77-4.49 |  | 0.86 | 0.62-1.18 |  | 0.92 | 0.62-1.34 |
| 25 |  | REF |  |  | REF |  |  | REF |  |  |  |  |
| 30 |  | 0.97 | 0.86-1.09 |  | 0.70 | 0.57-0.85 |  | 1.12 | 0.88-1.44 |  | 1.07 | 0.80-1.44 |
| 40 |  | 0.91 | 0.64-1.28 |  | 0.81 | 0.47-1.39 |  | 1.42 | 0.68-2.97 |  | 1.22 | 0.51-2.96 |
| Smoking |  |  |  |  |  |  |  |  |  |  |  |  |
| Never regular smoker |  | REF |  |  | REF |  |  | REF |  |  | REF |  |
| Former regular smoker |  | 2.09 | 1.55-2.80 |  | 1.30 | 0.81-2.07 |  | 1.27 | 0.73-2.18 |  | 2.38 | 1.07-5.30 |
| Current smoker |  | 2.13 | 1.51-3.01 |  | 2.95 | 1.65-5.25 |  | 3.26 | 1.65-6.44 |  | 8.32 | 3.48-19.89 |
| Diabetes |  | 3.25 | 2.39-4.42 |  | 1.48 | 0.91-2.41 |  | 1.12 | 0.57-2.22 |  | 2.03 | 0.98-4.21 |
| Cardiovascular disease |  | 4.94 | 3.65-6.70 |  | 1.34 | 0.83-2.16 |  | 1.55 | 0.82-2.93 |  | 1.25 | 0.59-2.67 |
| Hypertension |  | 2.90 | 2.25-3.74 |  | 1.35 | 0.89-2.06 |  | 1.01 | 0.61-1.69 |  | 1.43 | 0.81-2.53 |
| Grip strength |  |  |  |  |  |  |  |  |  |  |  |  |
| Lowest third |  | 2.20 | 1.62-3.00 |  | n/a |  |  | n/a |  |  | n/a |  |
| Middle third |  | 1.30 | 0.92-1.83 |  | n/a |  |  | n/a |  |  | n/a |  |
| Highest third |  | REF |  |  | n/a |  |  | n/a |  |  | n/a |  |

^a^Non-linearity of the association for eGFR, age and body mass index was modelled as specified in supplementary methods. ^b^Adjusted for age; sex; ethnicity; body mass index; smoking and self-reported pre-existing diagnoses of cardiovascular disease, diabetes and hypertension. Stratified into thirds of the distribution of grip strength standardized for age and sex. ^c^No events. Abbreviations: eGFR, estimated glomerular filtration rate in ml/min/1.73 m^2^ body surface area according to the CKD-EPI equation; OR, odds-ratio; 95% CI, 95% confidence interval; n/a, not applicable.

| **Supplementary table 2.** Associations between estimated glomerular filtration rate and all-cause mortality. Sensitivity analyses with additional adjustment for chronic obstructive pulmonary disease, cancer or congestive heart failure. | | | | | | | | | | | | | | | | | | | | |
| --- | --- | --- | --- | --- | --- | --- | --- | --- | --- | --- | --- | --- | --- | --- | --- | --- | --- | --- | --- | --- |
|  | **Adjusted model including COPD^ab^** | | | | | |  | **Adjusted model including cancer^ac^** | | | | | |  | **Adjusted model including CHF^ad^** | | | | | |
|  | **Thirds of grip strength** | | | | | |  | **Thirds of grip strength** | | | | | |  | **Thirds of grip strength** | | | | | |
|  | **Lowest third** | | **Middle third** | | **Highest third** | |  | **Lowest third** | | **Middle third** | | **Highest third** | |  | **Lowest third** | | **Middle third** | | **Highest third** | |
| **eGFR** | **OR** | **95% CI** | **OR** | **95% CI** | **OR** | **95% CI** |  | **OR** | **95% CI** | **OR** | **95% CI** | **OR** | **95% CI** |  | **OR** | **95% CI** | **OR** | **95% CI** | **OR** | **95% CI** |
| 37.5 | 2.30 | 1.42-3.72 | 0.95 | 0.39-2.31 | 0.84 | 0.30-2.37 |  | 2.28 | 1.42-3.67 | 0.95 | 0.39-2.31 | 0.83 | 0.30-2.34 |  | 2.24 | 1.39-3.61 | 0.98 | 0.40-2.39 | 0.84 | 0.30-2.37 |
| 52.5 | 1.52 | 1.20-1.94 | 0.96 | 0.51-1.82 | 0.88 | 0.42-1.85 |  | 1.52 | 1.19-1.93 | 0.96 | 0.51-1.82 | 0.88 | 0.42-1.83 |  | 1.51 | 1.18-1.92 | 0.98 | 0.52-1.86 | 0.89 | 0.42-1.85 |
| 75.0 | 1.13 | 1.05-1.20 | 0.98 | 0.76-1.27 | 0.95 | 0.71-1.28 |  | 1.12 | 1.05-1.20 | 0.98 | 0.76-1.27 | 0.95 | 0.71-1.27 |  | 1.12 | 1.05-1.20 | 0.99 | 0.77-1.28 | 0.95 | 0.71-1.28 |
| 90.0 | REF |  | REF |  | REF |  |  | REF |  | REF |  | REF |  |  | REF |  | REF |  | REF |  |
| 97.5 | 0.96 | 0.93-0.98 | 1.01 | 0.89-1.14 | 1.03 | 0.88-1.19 |  | 0.96 | 0.93-0.98 | 1.01 | 0.89-1.14 | 1.03 | 0.89-1.19 |  | 0.96 | 0.93-0.98 | 1.00 | 0.88-1.14 | 1.02 | 0.88-1.19 |
| 112.5 | 0.89 | 0.83-0.95 | 1.02 | 0.70-1.50 | 1.08 | 0.69-1.68 |  | 0.89 | 0.83-0.95 | 1.02 | 0.70-1.50 | 1.08 | 0.70-1.68 |  | 0.89 | 0.83-0.95 | 1.01 | 0.69-1.48 | 1.08 | 0.69-1.67 |

^a^Adjusted for age; sex; ethnicity; body mass index; smoking and self-reported pre-existing diagnoses of cardiovascular disease, diabetes and hypertension. Stratified into thirds of the distribution of grip strength standardized for age and sex. Non-linearity of the association for eGFR, age and body mass index was modelled as specified in supplementary methods.
^b^Additional adjustment for pre-existing COPD. ^c^Additional adjustment for pre-existing cancer. ^d^Additional adjustment for pre-existing CHF. Abbreviations: OR, odds-ratio; 95% CI, 95% confidence interval; eGFR, estimated glomerular filtration rate in ml/min/1.73 m^2^ body surface area according to the CKD-EPI equation; COPD, chronic obstructive pulmonary disease; CHF, congestive heart failure.

| **Supplementary table 3.** Associations between estimated glomerular filtration rate and all-cause mortality. Sensitivity analyses not excluding individuals with eGFR outside the range of 15-120 ml/min/1.73 m^2^ body surface area. | | | | | | | | |
| --- | --- | --- | --- | --- | --- | --- | --- | --- |
|  | **Adjusted^a^ model stratified by grip strength** | | | | | | | |
|  | **Lowest third** | |  | **Middle third** | |  | **Highest third** | |
| **eGFR** | **OR** | **95% CI** |  | **OR** | **95% CI** |  | **OR** | **95% CI** |
| 37.5 | 2.33 | 1.39-3.92 |  | 1.09 | 0.47-2.55 |  | 0.84 | 0.30-2.36 |
| 52.5 | 1.61 | 1.20-2.15 |  | 1.06 | 0.58-1.95 |  | 0.88 | 0.42-1.84 |
| 75.0 | 1.16 | 1.06-1.27 |  | 1.03 | 0.80-1.31 |  | 0.95 | 0.71-1.28 |
| 90.0 | REF |  |  | REF |  |  | REF |  |
| 97.5 | 0.94 | 0.91-0.98 |  | 0.99 | 0.87-1.11 |  | 1.03 | 0.88-1.19 |
| 112.5 | 0.85 | 0.77-0.94 |  | 0.96 | 0.67-1.38 |  | 1.08 | 0.69-1.67 |

^a^Adjusted for age; sex; ethnicity; body mass index; smoking and self-reported pre-existing diagnoses of cardiovascular disease, diabetes and hypertension. Stratified into thirds of the distribution of grip strength standardized for age and sex. Non-linearity of the association for eGFR, age and body mass index was modelled as specified in supplementary methods.
Abbreviations: OR, odds-ratio; 95% CI, 95% confidence interval; eGFR, estimated glomerular filtration rate in ml/min/1.73 m^2^ body surface area according to the CKD-EPI equation.

**
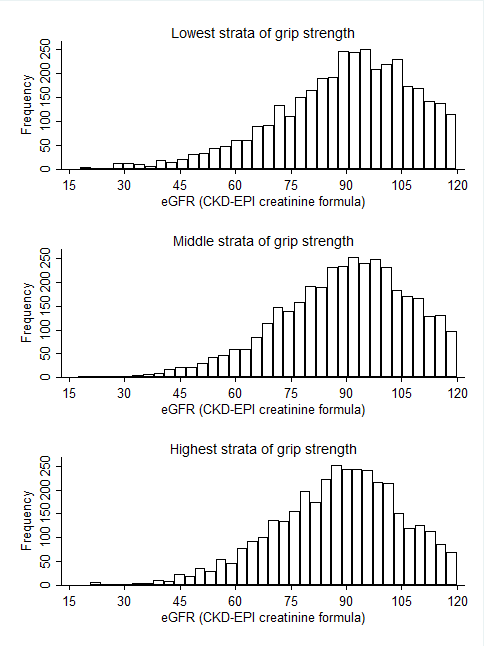
**

**Supplementary figure 1.** Distribution of estimated GFR from serum creatinine using the CKD-EPI equation, in three strata of grip strength. Stratification is based on thirds of the distribution of grip strength after standardisation by age and sex.
